# Some Erroneous Ideas of Ptomaines

**Published:** 1893-09

**Authors:** 


### Some Erroneous Ideas on Ptomaines.

Considerable misconception—not to say fundamental ignorance—is prevalent on the subject of ptomaines. This may clearly be seen from letters and articles which have appeared in the press since the recent sardine poisoning case, and also from statements made by medical men under conditions calculated to lend authority to their utterances. A case recently tried in a northern town, and an inquest held only a few miles from the same town, have brought out assertions regarding ptomaines which are almost as erroneous as the announcement in a popular weekly review that "ptomaines are bacteria." As, for obvious reasons, the erroneous statements made in connection with this subject are extremely dangerous, since they may lure the public into a feeling of safety or comfortable responsibility, it is desirable to point out some of the commoner and, at the same time more dangerous, misconceptions in regard to these poisons.

We hear it stated sometimes that all ptomaines are destroyed by such an amount of heat as is employed in the ordinary process of cooking. This might be so, if it were the case that ptomaines were bacteria; but if it were so, cases of food poisoning would be unheard of. Any one acquainted with Brieger's work and the chemistry of these toxines knows how stable and resistant against heat almost all, and especially the more important, ptomaines are; and that, therefore, there can be no safety in boiling, so far as the destruction of these bodies is concerned. Moreover, in the separation of many of these substances heat is constantly employed without the slightest effect on their toxic properties. Analogous observations are almost daily made during the preparation of the chemical products from pure cultures of bacteria, where heat is constantly used to destroy the micro organisms. If there be death in the pot, generally speaking no amount of boiling will prevent it. Another statement which invites unqualified dissent is that by boiling the poison will be distilled out and so much diluted by the water used as to become inocuous. Most ptomaines—there are exceptions—are very soluble in water, but it is absurd to assume that if hidden in the centre of a piece of meat they would diffuse through the tissues and be dissolved and diluted. Lastly, the fact that of several people who have partaken of a tainted article of food only one has suffered, has been used as an argument against the toxic nature of such food in any particular case. Can anything be more erroneous? The poison may be extremely localized in a particular piece of meat, the rest being free from it; and when we have to deal with a number of fish packed in the same tin, some only may be tainted, as was well shown in the case investigated by Dr. Stevenson, as here, moreover, the boiling oil in which the sardines are placed had not destroyed the lethal substance.

The prevention of food poisoning is a problem of extreme difficulty. In the meantime, however, we must remain fully alive to all the results of accurate chemical and physiological observations, and not lull ourselves into a feeling of safety while we may be face to face with death, all the more deplorable as it is unforseen accidental, but fortunately rare.—*British Medical Journal.*

